# Aerodynamic characteristics and genesis of aggregates at Sakurajima Volcano, Japan

**DOI:** 10.1038/s41598-022-05854-z

**Published:** 2022-02-07

**Authors:** M. C. Diaz Vecino, E. Rossi, V. Freret-Lorgeril, A. Fries, P. Gabellini, J. Lemus, S. Pollastri, A. P. Poulidis, M. Iguchi, C. Bonadonna

**Affiliations:** 1 Département des Sciences de la Terre, Université de Genève, Geneva, Switzerland; 2 Dipartimento di Scienze della Terra, Università di Firenze, Florence, Italy; 3 Institute of Environmental Physics, University of Bremen, Bremen, Germany; 4 Disaster Prevention Research Institute, Kyoto University, Kagoshima, Japan

**Keywords:** Solid Earth sciences, Volcanology

## Abstract

Aggregation of volcanic ash is known to significantly impact sedimentation from volcanic plumes. The study of particle aggregates during tephra fallout is crucial to increase our understanding of both ash aggregation and sedimentation. In this work, we describe key features of ash aggregates and ash sedimentation associated with eleven Vulcanian explosions at Sakurajima Volcano (Japan) based on state-of-the-art sampling techniques. We identified five types of aggregates of both Particle Cluster (PC) and Accretionary Pellet (AP) categories. In particular, we found that PCs and the first and third type of APs can coexist within the same eruption in rainy conditions. We also found that the aerodynamic properties of aggregates (e.g., terminal velocity and density) depend on their type. In addition, grainsize analysis revealed that characteristics of the grainsize distributions (GSDs) of tephra samples correlate with the typology of the aggregates identified. In fact, bimodal GSDs correlate with the presence of cored clusters (PC3) and liquid pellets (AP3), while unimodal GSDs correlate either with the occurrence of ash clusters (PC1) or with the large particles (coarse ash) coated by fine ash (PC2).

## Introduction

Aggregation processes play a significant role in the sedimentation and transport of fine ash from volcanic plumes^1,2^. Dispersal and deposition of volcanic ash can impact human and livestock health and affect local and international socio-economic systems^3–7^. Constraining the dynamics of aggregation processes in volcanic plumes and clouds is key to reduce the impact of volcanic eruptions^8–10^.

Ash aggregates are usually classified according to Brown et al.^1^ and Bagheri et al.^11^ who describe them in terms of Particle Clusters (PC), and Accretionary Pellets (AP). Particle Clusters (PC) are loosely bound aggregates that are thought to be mostly held together by electrostatic forces and they do not usually require the presence of a liquid phase as a binding mechanism (PCs include PC1: ash clusters, PC2: coated particles, and PC3: cored clusters)^1,11,12^; they rarely survive impact with the ground. On the other hand, Accretionary Pellets (AP) are typically associated with wet aggregation (AP1: poorly structured pellets; AP2: pellets with concentric structures; AP3: liquid pellets)^1^; in particular, the AP2 type (i.e., accretionary lapilli) is more easily found in deposits due to a higher preservation potential^13,14^. PC1s are irregular aggregates formed only by fine ash; PC2s consist of a large particle (crystal, pumice or lithic clast) partially covered with fine ash; PC3s consist of a core particle covered by a thick shell of particles < 90 µm ^1,11^; AP1s are spherical to sub-spherical aggregates with poor internal structure; AP2s (also defined as accretionary lapilli) are spherical to sub-spherical to cylindrical aggregates characterized by a pronounced internal structure (i.e., concentric layers); AP3s are rain drops filled with ash particles. The aerodynamic behavior of both PC and AP types plays an important role in modifying the residence time of ash in the atmosphere. It is, therefore, essential to collect field observations that can provide a full description of key aggregate properties such as size, shape and density that can best inform numerical models used for ash forecasting^11,14,15,16–18^. In particular, the studies of Bagheri et al.^11^ and Gabellini et al.^17^ have shown that high-speed camera (HSC) videos of falling aggregates, combined with simultaneous sample collection on adhesive paper for further analysis at the Scanning Electron Microscope (SEM) can provide an accurate description of the overall internal structure of the object, even for aggregates that are typically not preserved in the deposit (e.g., the PC type). This represents the current state-of-the-art of the sampling techniques for the observation of ash aggregates in the field. We applied this methodological approach to investigate the occurrence of aggregates during eleven Vulcanian eruptions at Sakurajima volcano (Japan) in November 2019 that occurred under different meteorological conditions. At the same location of the HSC videos, we also collected tephra in various dedicated trays in order to measure the sedimentation rate and derive the associated grainsize distribution (GSD). Finally, meteorological modelling was carried out to best correlate aggregate typology with meteorological conditions.

### Regional setting

**Figure 1:**
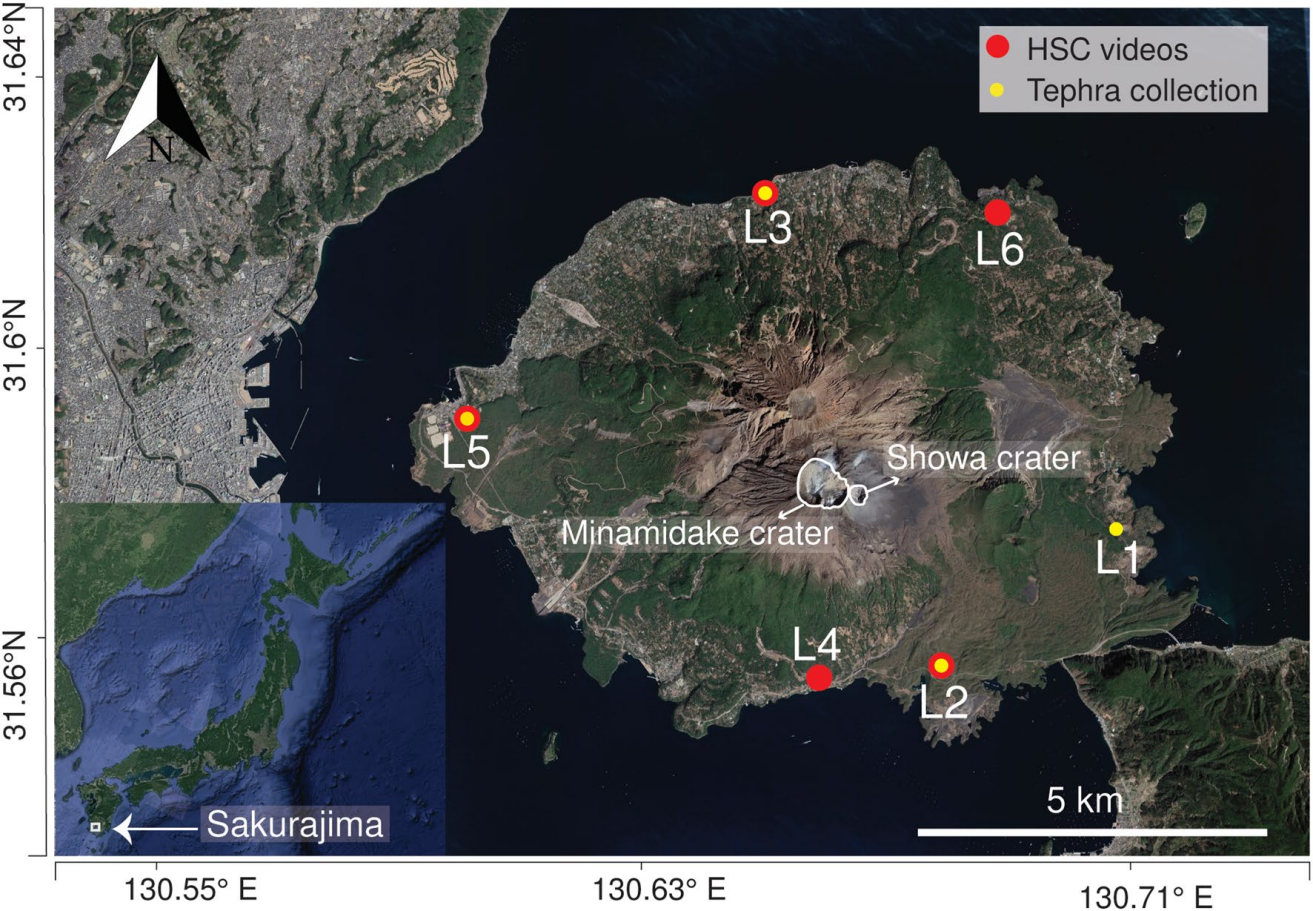
Location of the study area. The circles correspond to individual locations; L1 corresponds to location used on November 15, L2 was used on November 16, L3 was used on November 17, L4 was used on November 20, L5 was used on November 23 and L6 was used on November 20 (see Table 1 for more details). Large red circles represent the locations of the HSC videos and small yellow circles represent the location of tephra collection. Image taken from Google Earth (03/03/2017). *Image © 2021 Maxar Technologies*.

Sakurajima volcano is a post caldera volcano situated in the northern part of Kagoshima Bay, Japan, well known for its Vulcanian eruptions (Fig. 1); in fact, it represents an excellent natural laboratory for studying volcanic sedimentation because of its persistent activity^19–21^. It consists of three central cones: Kitadake, Nakadake and Minamidake^22^. Vulcanian activity started in 1955 at the Minamidake crater and is still persisting at the time of writing (June 2021), with almost daily Vulcanian explosions occurring mainly at the Minamidake crater and sometimes at the Showa crater.

Ash aggregation at Sakurajima volcano has been first observed by Tomita et al.^23^ as wet aggregates falling from a water rich eruption plume, as well as by Gilbert et al.^24^ and Sparks et al. ^25^ that reported ash clusters < 3000 µm made of particles < 200 µm. In addition, Miwa et al.^18^ investigated the sedimentation processes from HSC images taken during a Vulcanian eruption in 2016. In particular, they observed a secondary population within the GSD of tephra samples associated with the presence of ash aggregates, as well as low density aggregates (i.e., ash clusters). In our field campaign, we observed the presence of five different types of aggregates (e.g., Ash Clusters—PC1, Particle Clusters—PC2, Cored Clusters—PC3, Poorly Structured Pellets—AP1 and Liquid Pellets—AP3), associated with similar eruptive dynamics (i.e., plume height), but variable meteorological conditions (i.e., relative humidity and presence of rain). The diversity of aggregates found motivated us to further investigate and discuss the relationship between each aggregate type, the internal GSD of the aggregates and the external atmospheric conditions, which represents one of the most innovative aspect of this work. The analyzed aggregates were sampled at different locations within 5 km from the vent and were associated with eruptions that occurred between 15 and 26 November 2019. Aggregates are described in terms of their aerodynamic properties (i.e., terminal velocity and density) and correlated to specific sedimentation features (i.e., accumulation rate) and atmospheric conditions (i.e., Relative Humidity or RH and occurrence of rain). The HSC videos and SEM analyses, combined with plume observations and atmospheric data, allowed us to correlate and explain the different observed aggregates depending on the meteorological conditions, that may have preferentially led to the formation of APs or PCs. PC aggregates were observed and identified using both the HSC videos and the SEM tapes, while AP aggregates were only identified based on SEM tapes.

## Results

### Eruption characterization

We studied eleven different Vulcanian eruptions during eleven days of field investigations at Sakurajima volcano, Japan (15–26 November 2019). All the sampling locations were situated between 2.6 and 5.2 km from the vent (Minamidake summit crater) (Fig. 1). The locations are distributed around the volcano as the collection was done as close as possible to the dispersal axis that changed according to the wind conditions. The Japan Meteorological Agency (JMA) classified the volcanic activity level during the sampling period as moderately high. Information on the eruptions investigated along with the meteorological conditions, types of data collected, and types of aggregates identified are summarized in Table 1. Information on meteorological conditions (temperature, relative humidity, presence of rain) was gathered at the sampling site using a weather station (See “Methods”).

**Table 1:** Sampling locations, time of eruptions given in JST (offset UTC + 9:00 h), eruptive conditions and meteorological conditions; NA indicates not available. Information on the type of data collected and types of aggregates identified is also provided. RH indicates Relative Humidity. Plume height (in meters above sea level) was extracted both from the dataset published by the Japan Meteorological Agency and from High-Definition videos (see “Methods” section for more details); some plume height information is missing due to bad weather conditions.

Eruption ID	Eruption date and time (JST)	Plume height (m.a.s.l)	Sampling location	Distance from the vent (km)	Atm. T (°C)	Atm. RH (%)	Rain	Type of data collected	Aggregate types
A	Nov 15, 17:09	2700	L1	4.3	12.4	74.6	No	Tray collection	PC3
B	Nov 16, 13:49	2422	L2	3.0	20.0	41.8	No	Tray collection, HSC videos	PC2
C	Nov 16, 14:21	NA	L2	3.0	NA	NA	No	HSC videos, SEM tapes	PC2
D	Nov 16, 15:12	2236	L2	3.0	NA	NA	No	HSC videos	PC2
E	Nov 16, 16:06	NA	L2	3.0	NA	NA	No	Tray collection, SEM tapes	PC2
F	Nov 17, 11:12	1780	L3	4.5	22.8	61.6	No	HSC videos	PC2, PC1, PC3
G	Nov 17, 13:52	3300	L3	4.5	20.9	62.5	No	Tray collection, HSC videos, SEM tapes	PC1, PC2, PC3
H	Nov 20, 10:56	2792	L4	2.6	14.0	54.0	No	SEM tapes	PC2
I	Nov 23, 14:20	3100	L5	5.2	21.3	75.2	Yes	Tray collection, HSC videos, SEM tapes	AP1, AP3, PC1, PC2
J	Nov 23, 16:11	2200	L5	5.2	20.9	75.6	Yes	Tray collection, HSC videos	PC1, AP3
K	Nov 26, 14:24	3700	L6	4.9	18.8	73.0	Yes	HSC videos, SEM tapes	AP3, PC1, PC2, PC3

### Types of aggregates collected during the field investigations

**Figure 2:**
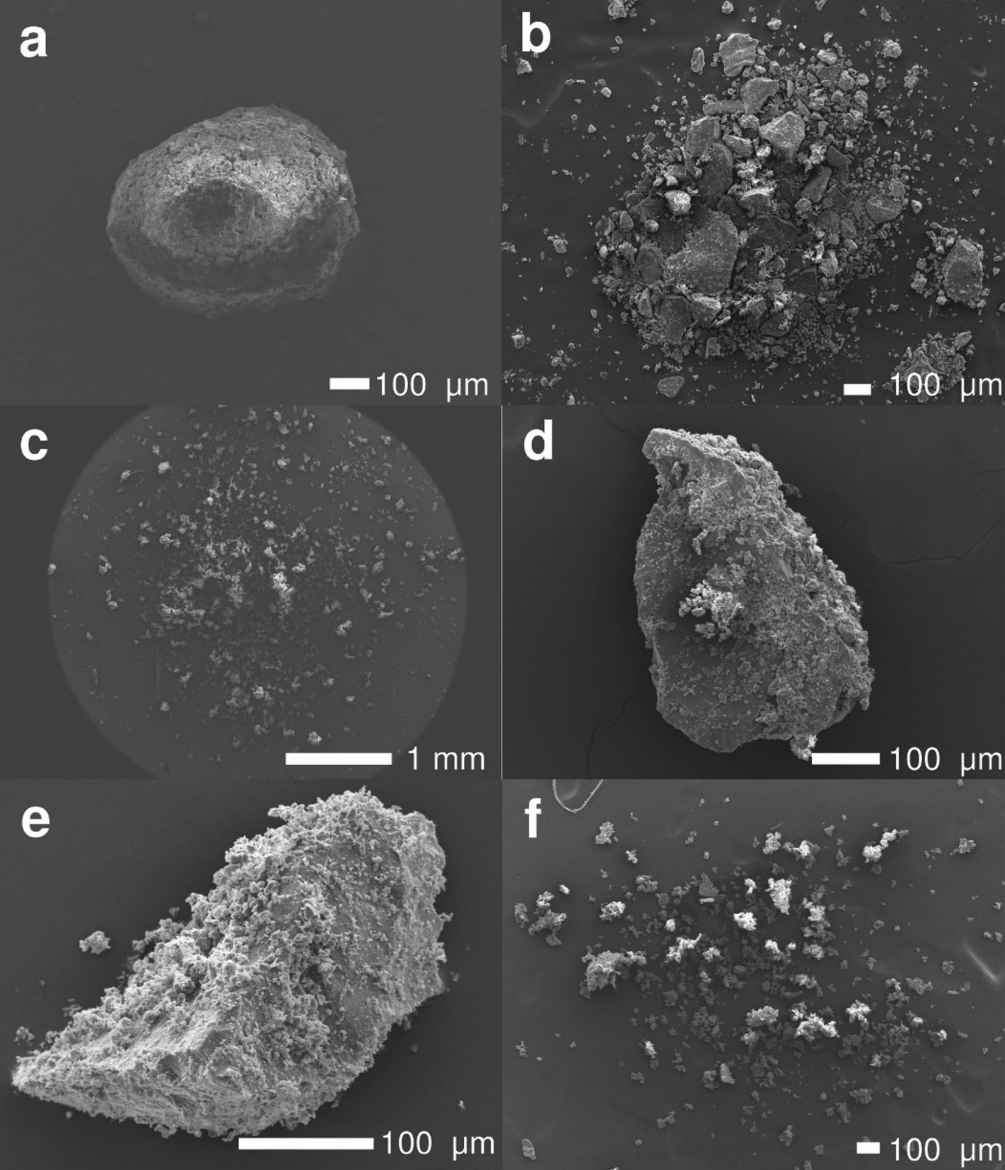
SEM images of the different types of aggregates collected. (**a**) AP1 from eruption I collected at L6; (**b**) AP3 from eruption K collected at L7; (**c**) PC1 from eruption I collected at L6; (**d**) and (**e**) PC2s from eruption C collected at L6; and (**f**) shell of PC3 from eruption K collected at L7.

Five types of aggregates were identified associated with eleven eruptions using observations from the HSC videos along with SEM analyses of the samples collected on adhesive papers (Table 1 and Fig. 2). The occurrence of Particle Clusters (i.e., PC1s, PC2s and PC3s) was confirmed using the critical interpretation of aerodynamic data (i.e., terminal velocity and density). The occurrence of Accretionary Pellets (i.e., AP1s and AP3s) was confirmed using the SEM images as HSC videos were not available.

We identified the following aggregates:

- AP1 (or poorly-structured accretionary pellets of Brown et al.^1^) during eruption I (Fig. 2a); this type of aggregate was identified based on associated sub-spherical shape with diameters of approximately 500 to 600 μm.
- AP3 (or liquid pellets of Brown et al.^1^) during eruptions I, J and K; these aggregates consist of liquid water drops containing ash particles. We observed sizes ranging from 590 µm to 2400 µm.
- PC1 (or ash clusters of Brown et al.^1^) during eruptions G, I, J and K; these are irregular aggregates that mostly consist of fine ash with no evident internal core. The observed aggregates vary from 275 µm to 944 µm.
- PC2 (or coated particles of Brown et al.^1^) during eruptions B, C, D, E, F, H and K; these aggregates consist of coarse-ash particles partially covered with fine ash that usually fall off upon impact with the ground or with the adhesive tape. We observed sizes that ranged from 220 to 493 μm.
- PC3 (or cored clusters of Bagheri et al.^11^) during eruptions A, G and K; these aggregates are characterized by the presence of a coarse particle (i.e., core) covered by a thick shell of fine ash. PC3 aggregates could only be identified with the HSC videos due to their low preservation potential; their size ranges between 260 µm and 584 µm.

It is important to notice that when the Relative Humidity (RH) was greater than 70% and there was presence of rain during fallout (e.g., eruptions I, J and K), we found Accretionary Pellets such as AP1s (poorly structured pellets) and AP3s (liquid pellets). In contrast, Particle Clusters (i.e., PC1s, PC2s and PC3s) were identified in all meteorological conditions (Table 1). For example, we identified PC3 aggregates (cored clusters) during eruption A for which the RH values were higher than 70% and without rain, as well as in eruption K with RH > 70% and rain and in eruptions F and G with RH ∼62% and no rain. In addition, PC2 aggregates (coated particles) were found during eruptions with no rain and low RH (e.g., eruption B, F, or H), as well as during eruptions with rain and high RH (eruption I and K). Finally, PC1 aggregates (ash clusters) were found associated with eruptions with RH > 61.6%, regardless of the presence or absence of rain (e.g., eruptions F, G, I, J and K).

### Aggregation dynamics associated with different aggregate types

**Figure 3:**
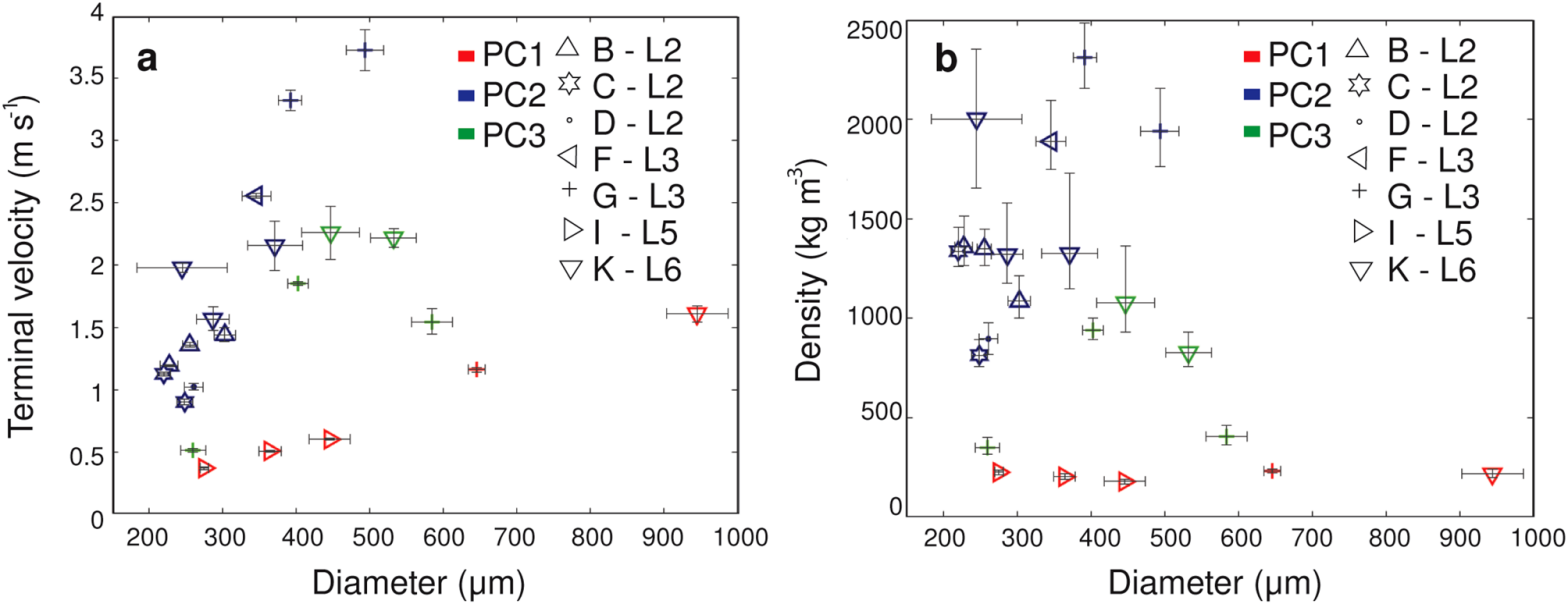
Aerodynamic properties of aggregates (PC1s in red, PC2s in blue and PC3s in green) analyzed with the HSC videos. (**a**) Terminal velocity versus size and (**b**) aggregate density versus size.

HSC videos provide information on the aerodynamic properties of the falling aggregates. The image processing of HSC videos allowed us to obtain the terminal velocity, size and density of the observed aggregates (see “Methodology” section) (Fig. 3). In total, 22 aggregates associated with 7 different eruptions were analyzed and classified according to the classification of Brown et al.^1^ and Bagheri et al.^11^ as PC1, PC2 and PC3. The size of all the PC aggregates investigated ranges between 220 and 944 μm, the terminal velocity varies between 0.3 and 3.7 m s^−1^and density values vary between 179 and 2311 kg m^−3^. It should be mentioned that the tephra produced by Sakurajima volcano has a dense rock equivalent (DRE) density between 2500 and 2700 kg m^−3^ ^11,26^. PC1s show low terminal velocities (0.3–1.6 m s^−1^) and low densities (179–232 kg m^−3^) and represent about 20% of the aggregates analyzed. PC2s are associated with terminal velocities of 0.9–3.7 m s^−1^ and densities of 815–2311 kg m^−3^, with the smallest aggregates (< 350 μm) showing terminal velocities < 2 m s^−1^; these types of aggregates represent the most abundant type of all aggregates analyzed (> 50%). PC3s show terminal velocities of 0.5–2.2 m s^−1^ and densities of 347–1000 kg m^−3^ and represent 17% of all aggregates analyzed. PC3 aggregates show lower density values than PC2 aggregates of similar sizes.

### Grainsize of tephra deposits and individual aggregates

**Figure 4:**
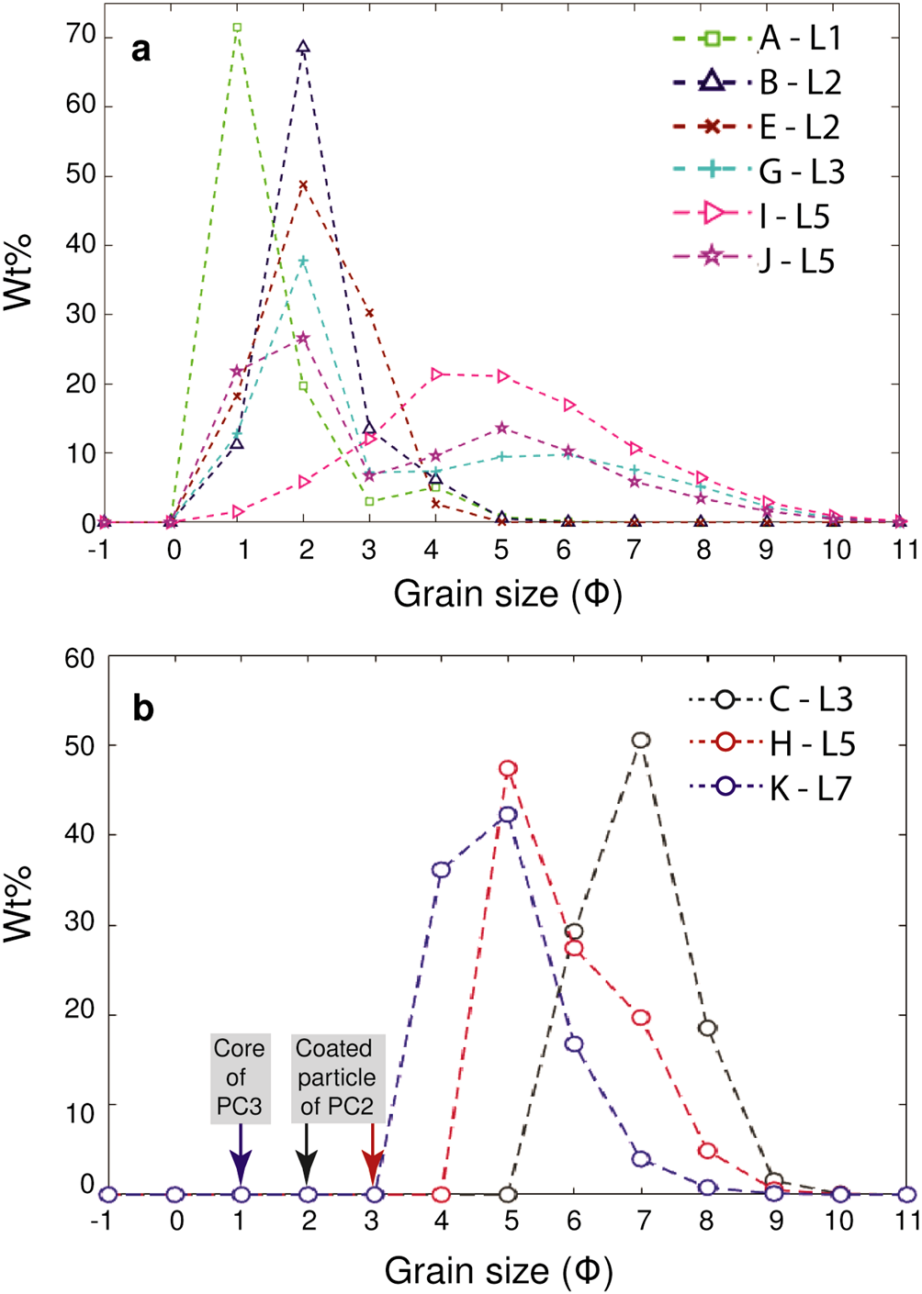
(**a**) GSDs in weight percentage (Wt.%) of tephra collected in trays (bulk samples) representative of the whole time of sedimentation at each location; (**b**) GSD of individual aggregates collected on SEM adhesive tape: coating of two PC2 aggregates from eruption C collected at L3 and eruption H collected at L5 and shell of one PC3 aggregate from eruption K collected at L7. The position of the gray and red arrows indicates the size of the coated particles of eruption C and H (i.e., 325 μm and 158 μm–2 and 3 Φ, respectively) and the blue arrow corresponds to the core of PC3 of eruption K (i.e., 523 μm–1Φ).

The tephra samples collected at the same location as HSC videos, show both unimodal and bimodal distribution (Fig. 4a). In particular, eruptions B, E and I are characterized by a unimodal GSD with a mode at 2 Φ (B and E) and 4 Φ (I) (Φ =  − ${{\log}}_{{2}} {\text{(d}})$, while A, G and J are characterized by a bimodal GSD with two modes at 2 and 4 Φ, at 2 and 6 Φ and 2 and 5 Φ, respectively. Different aggregate types have been observed. Eruptions B and E are mostly associated with PC2, while most types (PC1, PC2, AP1 and AP3) have been observed during eruption I. In contrast, eruption A is associated with PC3, eruption G with PC1, PC2 and PC3 and eruption J with PC1 and AP3.

Furthermore, we calculated the GSD of the coating of two PC2s and the shell of one PC3 collected with the SEM adhesive tapes from three different eruptions: C, H and K (Fig. 4b). The coating of PC2 from eruption C has a mode at 7 Φ and the diameter of the coated particle was measured at 2 Φ. The coating of the other PC2 from eruption H has mode at 5 Φ and the diameter of the coated particle was measured at 3 Φ. Finally, the shell of PC3 from eruption K has a mode at 5 Φ and the associated core has a diameter of 1 Φ (calculated with the HSC videos because the core left the tape after the impact).

### Accumulation rate and occurrence of different aggregate types

**Figure 5:**
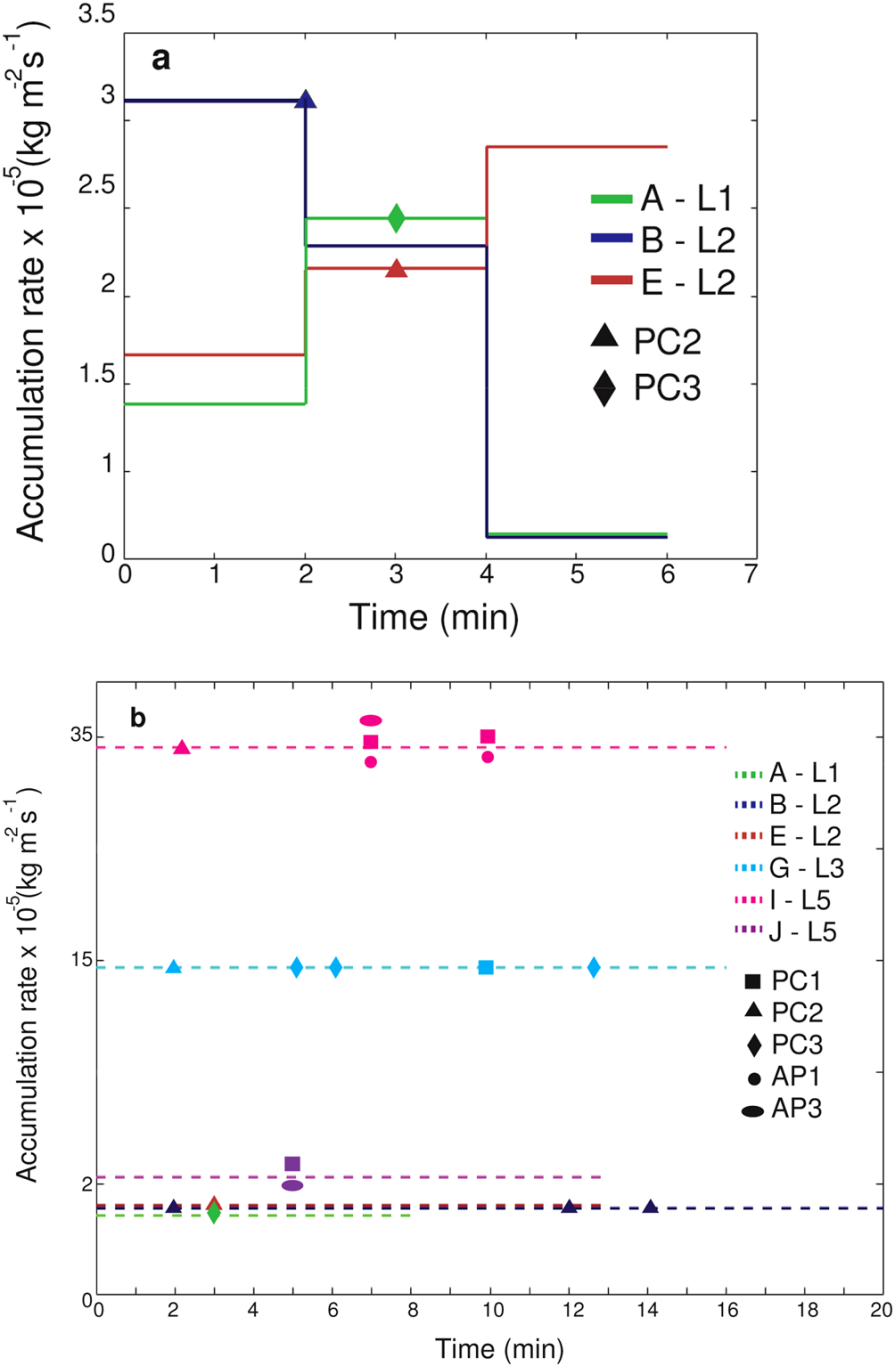
Accumulation rates as a function of time since the beginning of the fallout and timing of observed aggregate types. (**a**) Accumulation rate for three eruptions (A, B, E) based on multiple-tray collection during the same fallout and (**b**) average accumulation rate based on cumulative collection in a single tray for six eruptions (A, B, E, G, I and J). Different symbols indicate different aggregate types (PC1—square, PC2—triangle, PC3—rhombus, AP1—circle, AP3—ellipsoid). Time = 0 corresponds to the beginning of fallout at individual sites and beginning of ash accumulation in trays.

We analyzed both multiple trays and cumulative samples to determine the accumulation rate (Fig. 5). We defined time = 0 as the start of the fallout for every eruption in the corresponding location. The accumulation rate based on multiple trays is related to a maximum duration of 6 minutes and the individual trays are exposed for two minutes each (Fig. 5a). In contrast, the average accumulation rate is based on a cumulative sample collected in a single tray (Fig. 5b). Even though sampling locations are all at a similar distance from the volcano (2.6–5.2 km), fallout duration depends on eruption dynamic and is different for each eruption. In addition, given that one tray was used during the whole fallout to calculate the average accumulation rate, the duration shown in Fig. 5b is longer than in Fig. 5a. The temporal evolution of the accumulation rate is variable, showing very distinct trends among the considered eruptions. In fact, eruption E is associated with a gradual increase, while eruption B is associated with a gradual decrease and eruption A shows a peak value in the middle of fallout (i.e., 4 minutes from the start of the fallout) (Fig. 5a). The eruptions I and G are associated with the highest average accumulation rate of all the eruptions studied (33 × 10^–5^ kg m^−2^ s^−1^ and 15 × 10^–5^ kg m^−2^ s^−1^, respectively), while eruptions A, B, E, and J are characterized by an average accumulation rate ≤ 2 × 10^–5^ kg m^−2^ s^−1^ (Fig. 5b).

The studied eruptions not only are characterized by different accumulation rate, but also by different occurrence of aggregate types at different times. One PC3 aggregate was identified during eruption A after 3 min of the start of the fallout (green lines in Fig. 5). Eruption B and E are associated with PC2 aggregates; three PC2 aggregates were identified at 2, 12 and 14 minutes after the start of the fallout of eruption B and one PC2 aggregate was identified 3 minutes after the start of the fallout of eruption E (Fig. 5b). Interestingly, for eruption B, E, G and I the first phase of the fallout was characterized by the arrival of PC2 aggregates. In particular, the first arrival of PC2s during eruption G was identified 2 minutes after the start of fallout, followed by a major arrival of large PC3s and PC1s after 6 and 10 min from the fallout onset, respectively. Additionally, the middle phase of the fallout of eruptions I and J, both associated with rainy conditions, were dominated by the simultaneous arrival of both PC1 and AP3 aggregates. Eruption I was also associated with the occurrence of AP1.

## Discussion

**Figure 6:**
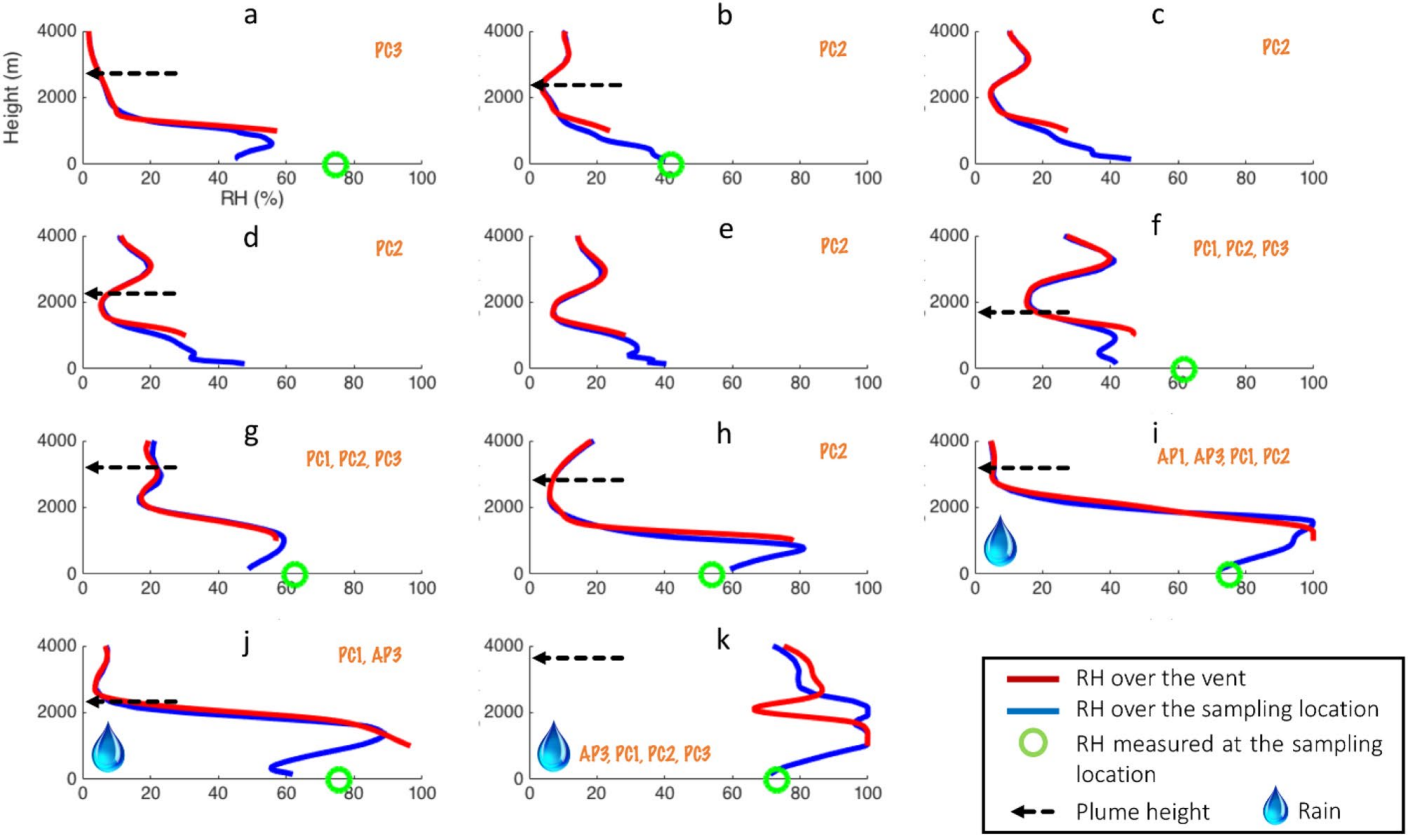
Modelled relative humidity profiles above the vent and above the sampling locations for all the eruptions obtained using the Weather Research and Forecasting (WRF) model. The green dot is the actual value measured using the portable meteorological station at the sampling location. The dotted arrow indicates the plume height associated with each eruption. All heights are shown above sea level. The raindrop indicates events characterized by rain fall. Identification label for all eruptions is shown on plots (a to k; Table 1).

Our observations confirm the efficiency of the multi-technique introduced by Bagheri et al.^11^ in providing a comprehensive and detailed description of ash aggregation in the field (i.e., combined use of HSC videos, SEM tapes and tephra collection in trays). It is important also to consider that the complexity of such a technique results in the analysis of a limited number of aggregates (density and terminal velocity characterization of 22 aggregates—Fig. 3, and GSD of 3 aggregates—Fig. 4), which, however, confirms the outcomes of Bagheri et al.^11^ and Gabellini et al.^17^ enlarging their datasets. Moreover, for the first time, this state-of-the-art approach has been applied to multiple eruptions that occurred with variable meteorological conditions (Fig. 6). The large variability of aggregate types presented with such a high temporal and spatial accuracy represents the major result of this work, since it has never been described by previous field studies. The observed variability in the relative abundancy of the aggregate types among the different eruptions can be correlated with the different atmospheric conditions, in particular with the different RH profiles and with the presence of rain (Table 1 and Fig. 6). It has already been observed that changing the RH of the air within the experimental system affects the rate of aggregate production^27^. However, Fig. 6 shows that poorly-structured accretionary pellets (AP1s) and liquid pellets (AP3s) have been identified only in the presence of liquid water (i.e., rain) regardless of the value of RH (eruptions I, J and K). It is also well known that accretionary lapilli (AP2s) are associated with the presence of liquid water^13,28,29^. However, the genesis of AP1s is less known^1^. According to Bonadonna et al.^15^ the formation of AP1s is associated with the availability of atmospheric moisture, while the presence of AP3s is mostly associated with the occurrence of rain drops that help to scavenge coarse‐ash particles. Our observations confirm this scenario; in fact, eruptions with relatively high values of RH but in the absence of liquid water did not trigger the formation of APs (eruptions A, G and H). Figure 6 also confirms that AP1s and AP3s only occurred in presence of atmospheric liquid water (eruptions I to K). On the other hand, PCs (i.e., ash clusters, coated particles or cored clusters) are found during all the eleven eruptions under analysis, regardless of the meteorological conditions. This is a key point that suggests that the binding mechanism for all the types of PCs is poorly correlated with RH and probably due to alternative sticking mechanisms, such as the presence of electrostatic charges on the surfaces of the particles. In fact, the independence of PCs on atmospheric RH indicates that the binding mechanism for these objects are most likely associated with fundamental processes that happen in the conduit or in the plume, more than with the external weather conditions. Among the processes known so far, fractoemission and triboemission are thought to play a key role, due to the fact that they can generate net electrostatic charges on the surface of the particles^24,30^. These processes always occur in explosive volcanic eruptions and provide a source of binding that does not depend on the external weather conditions. The coexistence of PCs and APs during the same rainy conditions (Fig. 6 eruption I, J, K) suggests that a large variety of different types of aggregates can be observed within the same eruption.

The analysis of the aerodynamic properties of the aggregates shows a broad range in their values of terminal velocity and density. Measured terminal velocities are between 0.3 and 3.7 m s^−1^ with the majority of the values < 2.0 m s^−1^. Values of terminal velocities of ash clusters (PC1) (0.3–1.6 m s^−1^) are consistent with experimental results of James et al.^31^ who report typical values of < 1 m s^−1^ for PC1 aggregates of sizes < 800 µm (their aggregation process was driven by electrostatic charges naturally imparted to the particles). In addition, associated density values of these experimental ash clusters (100–200 kg m^−3^) agree with the results of this study (i.e., values of 179 and 232 kg m^−3^ for aggregate diameters up to 944 μm). In fact, PC1s in our study mostly consist of particles < 40 μm with no core. Coated particles (PC2) are associated with the highest terminal velocities with density values between 815 and 2311 kg m^−3^ and sizes between 220 and 493 μm. The density values for PC3 aggregates are significantly lower than those of PC2 aggregates of similar sizes, which confirms the presence of a thick shell of fine ash on PC2 aggregates also observed in the HSC videos. All these values are in good agreement with the results of Bagheri et al.^11^ and Gabellini et al.^17^ for comparable eruptions of Sakurajima volcano, where Bagheri et al.^11^ studied a single explosion with a plume height of 2.8 km (a.s.l) and Gabellini et al.^17^ studied ten explosions during ten days of investigations with plume heights ranging between 1.5 km and 3 km (a.s.l); both investigations were carried out in August 2013. As an example, Gabellini et al.^17^ report density values between 250 and 2000 kg m^−3^ for the aggregates collected that coincide with the range of values obtained for the same type of aggregates (PC1, PC2 and PC3) during this study. The high settling velocities of the PC2s and PC3s explain the premature fallout of fine ash at proximal distances (< 5 km from the vent), which was also previously observed^11,16–18^.

**Figure 7:**
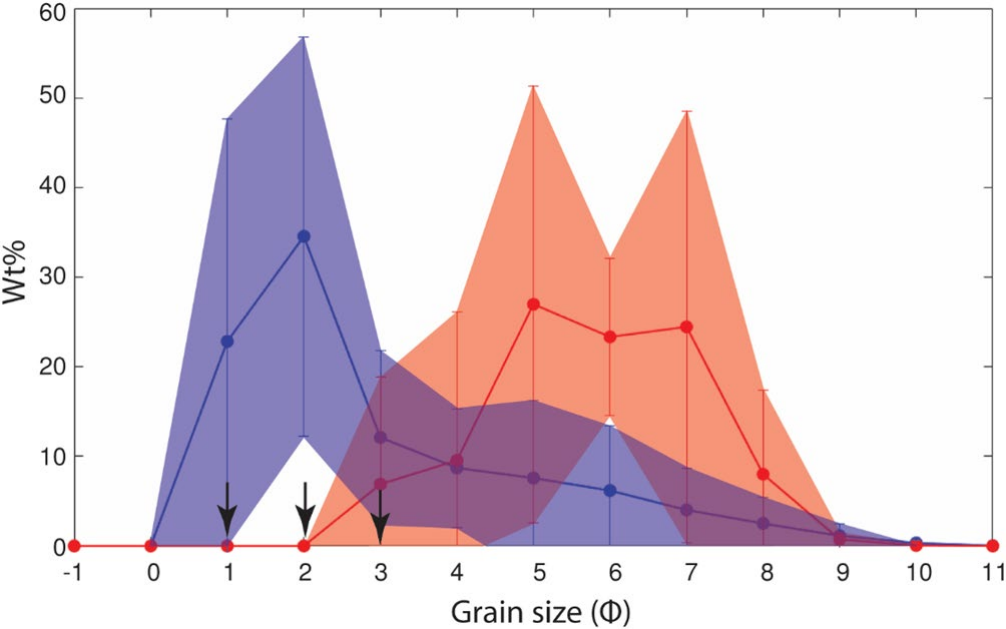
Comparison between the GSD of tephra samples from six eruptions (eruptions A, B, E, G, I and J; Fig. 4a) shown in blue, and the GSD of two types of aggregate (PC2 and PC3) determined from the SEM images for 3 eruptions (eruptions C, H and K; Fig. 4b), shown in red. The position of the arrows indicates the size of the coated particles that were 325 μm and 158 μm (2 and 3 Φ) and of the core of PC3 that was 523 μm (1Φ).

The GSD of tephra deposits has often been considered as an indication of the presence of aggregates^1^. In fact, bimodal GSDs of tephra deposits have often been interpreted as due to ash aggregation that increases the terminal velocity of fine ash particles^32,33^. In our investigation, bimodal GSDs are associated with eruptions A and G, that were characterized by the presence of cored clusters (PC3). These aggregates are composed of a coarse core (> 90 μm) and a thick shell of very fine material (< 90 μm) which explains the modes at 2 and 6 Φ (i.e., 250 μm and 16 μm respectively) for eruption G. Bagheri et al.^11^ reported this type of aggregates for the first time studying Sakurajima Vulcanian explosions and suggested that the polymodal GSD is associated with the deposition of cored clusters; they measured MdΦ^34^ for the GSD of PC3 aggregates between 4.5 and 5.5 Φ. This corroborates the idea that PC3s are very efficient at scavenging fine ash. Eruption J has also a bimodal GSD; however, in this case this distribution is associated with the presence of liquid pellets (AP3). The occurrence of AP3 is described in situations where the liquid drops (up to several millimeters) scavenge particles of different sizes and lead to sedimentation in the form of mud^35^. For instance, Brown et al.^1^ describe AP3 aggregates with a wide range of particle sizes from 0.1 to 1000 µm and, therefore, the bimodal GSD of this eruption corresponds to the expected size range of the aggregate identified. In contrast, unimodal GSDs from eruptions B and E are associated with coated particles (PC2). The size of the associated coated particles (185 to 493 μm) is consistent with the GSD mode at 1–2 Φ (250 to 500 μm) for both eruptions. PC2 aggregates have been associated with unimodal distribution in past investigations; for instance, Gabellini et al.^17^ describe GSD of tephra samples with modes peaked at 2.5 and 3 Φ for eruptions with identified coated particles. In addition, eruption I presents a unimodal GSD with a mode at 4-5Φ (31–125 μm), which is consistent with the occurrence of ash clusters (PC1) that are typically composed of particles of ~ 40 μm (according to Brown et al.^1^). In particular, Fig. 7 shows the GSD of the tephra samples from six eruptions in blue (eruptions A, B, E, G, I and J) and the GSD of the coating of the PC2 and the shell of PC3 aggregates collected with the adhesive tape from eruptions C, H and K in red; the size of the coated particles and the core of the PC3s are also shown (black arrows). The comparison between the two GSDs shows how the fine mode of tephra deposits, located at 5 Φ corresponds to the shell of PC3 and the coating of PC2.

Given that all sampling locations are at similar distance from the vent (2.6–5.2 km), measurements of the accumulation rates can be compared between eruptions and vary mostly as a function of the eruption dynamics (e.g. plume height) and wind dispersal. Furthermore, eruptions I, G and K that are associated with the highest plumes (> 3100 m.a.s.l) and the highest accumulation rates (33 × 10^–5^ kg m^−2^ s^−1^ and 15 × 10^–5^ kg m^−2^ s^−1^ for eruptions I and G, respectively; accumulation rate for eruption K is not available) are also associated with the largest diversity in aggregates identified (i.e., PC1, PC2, PC3, AP1 and AP3). Considering that, for a buoyant thermal, plume height is a monotonically increasing function of the erupted mass^36^, this may indicate that the production of different type of aggregates within a single eruption is related both to favorable atmospheric conditions for AP aggregates (i.e., liquid water) and to the residence time within the volcanic plume and cloud (assuming that a higher plume provides the opportunity for particles and aggregates to stay longer within the plume and cloud) as well as to the amount of ash available.

## Conclusions

Our results provide new insights into the occurrence of different types of aggregates as well as their aerodynamic characteristics during eruptions of similar eruptive dynamics but associated with variable meteorological conditions. In particular:

- We identified relationships between the meteorological conditions and the occurrence of different types of aggregates. Aggregates classified as Accretionary Pellets (AP1s and AP3s) were identified during eruptions with rain and RH > 70%. On the other hand, Particle Clusters (PCs) were associated with both dry and rainy atmospheric conditions (i.e. RH between 41.8% and 75.2%). In particular, we also found that PCs and APs can coexist in case of rainy conditions.
- Aerodynamic properties for Particle Clusters (PC) depend on the specific type (i.e., PC1, PC2 and PC3). PC1 aggregates show the lowest densities (179–232 kg m^−3^) and terminal velocities (0.3–1.6 m s^−1^) compared to the other aggregate types. In contrast, PC2 aggregates show the highest terminal velocities and densities, i.e., 0.9–3.7 m s^−1^ and 815–2311 kg m^−3^, respectively. PC3 aggregates have intermediate values of terminal velocities (0.5–2.2 m s^−1^) and densities (347–1000 kg m^−3^). Unfortunately, AP aggregates were not captured in HSC videos and their aerodynamic properties could not be determined.
- The tephra samples studied are associated with both unimodal and bimodal GSDs. Eruptions A, G and J show bimodal GSDs in relation to the presence of PC3 or AP3 aggregates. In contrast, the unimodal GSD of eruptions B, E and I agrees with the presence of mostly PC2 or PC1 aggregates.
- The eruptions with the highest plumes (I, G and K) and the highest accumulation rate (I and G) are associated with the largest diversity of aggregate types. High values of RH and the presence of rain also allow for the formation of AP1s and AP3s (eruption I and K). Interestingly to notice that, when present, PC2 are commonly observed during the first phase of the fallout.

## Methods

Our methodology is based on the simultaneous use of High-Speed Camera (HSC) videos, collection of aggregates on adhesive papers to be analyzed at the Scanning Electron Microscope (SEM) and collection of tephra in dedicated trays^11^ at specific locations (Table S1; Supplementary Material). The trajectories of the aggregates were filmed during their fall in the air and their impact with an adhesive paper. The evaluation of the sedimentation rate in dedicated trays was carried out at the same location where the HSC videos were taken. Eruptions are labelled with letters, from A to K (Table 1). Along with the equipment previously mentioned for the data collection, we used a Kestrel 5500 weather station to evaluate the meteorological conditions. The weather station was placed at the same location as the HSC and the trays; we collected basic meteorological information at the ground level, such as air temperature, relative humidity, and presence of rain. For some of the eruptions on 16 November (see Table 1), it was not possible to obtain the meteorological information due to technical problems.

In order to obtain complimentary profiles of relative humidity above the vent and above the sampling location for all days, meteorological simulations were carried out using the version 4.0 of the Weather Research and Forecasting (WRF) model^37^ (Tables S2 and S3; Supplementary Material). The plume heights associated with eruptions A, G, I, J and K were obtained from the dataset provided online by the Japan Meteorological Agency (https://www.jma-net.go.jp/kagoshima/vol/data/skr_exp_list/skr_exp_2019.html), whilst smaller plume heights associated with eruptions B, D, F, and H were determined from High-Definition videos. For eruptions A and G, both measurements from the Japan Meteorological Agency and High-Definition videos were available, yielding similar results with a maximum discrepancy of 200 m. Unfortunately, it was not possible to estimate the plume height for eruptions C and E that were masked by the presence of meteorological clouds. HSC videos are available for all eruptions except for eruptions A, E and H (examples of PC1, PC2 and PC3 aggregates can be found in Supplementary Material).

### Processing of High-Speed Camera videos of falling aggregates

The Phantom Pro HS Camera mounted with a Nikon 60 mm f/2.8 D AF Micro Nikkor lens was used at the observation sites (Fig. 1) to capture the falling aggregates using different resolutions such as 1024 × 512, 1024 × 384, 1920 × 768 and 1024 × 576 pixels at 2000, 2000, 1100 and 1500 fps, respectively. The HSC was pointed at a glass plate inclined at 30° at a distance of 30 cm and was equipped with a SEM-ready double side adhesive tape. Both the HSC and the thin sections holder were placed on the same support, in such a way that it was possible to collect the same ash aggregates that were filmed by the HSC (Fig. S1; Supplementary Material). We used a LED light when the conditions of light were not optimal (e.g., at night conditions), and we placed an optical diffuser behind the glass plate holder in order to obtain sharper images by homogenizing the light intensity of the background.

We collected more than 80 HSC videos of falling aggregates, but we chose 22 optimal HSC videos for this investigation based on various considerations. In fact, only aggregates that were in focus for at least three consecutive frames, that were not affected by lateral wind and that were falling at their terminal velocity were selected. To process the HSC videos, we converted them to 8-bit Tiff format images frame-by-frame, and we analyzed them with the software Fiji^38^ to extract information on shape parameters and position of the aggregates while falling to the adhesive tape (Table S4; Supplementary Material). With the previous information we calculated the diameter, terminal velocity and density of the aggregates; the associated uncertainties for these variables are found in section 6 of the Supplementary Material.

#### Diameter calculation

We used Eq. (1) to measure the diameter of the falling aggregates, ${\text{d}}_{{{\text{eq}}}} \left( {{\upmu m}} \right),$ employing the methodological approach proposed by Bagheri et al.^39^. This equation is used under the consideration that the aggregates have a smooth surface.

1 $${\text{d}}_{{{\text{eq}}}} = 0.928 \cdot \left( {{\text{L}} \cdot {\text{I}} \cdot {\text{S}}} \right)^{1/3}$$

The shape parameters L, I, and S (${\upmu m})$ describe the complex morphology of the aggregates^40^. L and I represent the maximum and minimum Feret diameter measured on the maximum projection of the area, and S represents the minimum Feret diameter measured on the minimum projection of the area.

#### Terminal velocity calculation

We calculated the terminal velocity, ${\text{V}}_{{\text{t}}} \left( {{\text{m s}}^{ - 1} } \right),$ for each falling aggregate using Eq. (2), considering the change in position along the y axis ${\text{y}}_{{{\text{i}} + 1}} - {\text{y}}_{{\text{i}}}$ and the change in time ${\text{t}}_{{{\text{i}} + 1}} - {\text{t}}_{{\text{i}}}$ from two consecutive frames ${\text{f}}_{{\text{i}}}$ and ${\text{f}}_{{\text{i + 1}}}$. To find the time interval amid frames we used the sampling rate of the respective video.

2 $${\text{V}}_{{\text{t}}} = \frac{{{\text{y}}_{{{\text{i}} + 1}} - {\text{y}}_{{\text{i}}} }}{{{\text{t}}_{{{\text{i}} + 1}} - {\text{t}}_{{\text{i}}} }}$$

To make sure that the aggregate was not accelerating and falling at its terminal velocity we compared the velocity between the first frame (when the particle entered the field of view) and last frame (before the aggregate collided with the plate) to the velocity between the last two frames before the collision. Furthermore, to make sure that the aggregate was not affected by lateral wind we used the value of the velocity in the x-axis to verify if it was < 10% of the velocity in the y-axis.

#### Density calculation

We calculated the aggregates densities ${\uprho }_{{{\text{agg}}}}$ (kg m^−3^) using Eq. (3) where ${\uprho }_{{\text{a}}}$ (kg m^−3^) is the density of the air, ${\text{C}}_{{\text{d}}}$ (no units) is the drag coefficient, ${\text{V}}_{{\text{t}}}$ (m s^−1^) is the terminal velocity and g (m s^−2^) is the gravity. The densities of the aggregates are derived from a non-linear relationship with the variables in Eq. (3) (i.e., Terminal velocity, shape, diameter, and air density—Table S6; Supplementary Material). Therefore, we used Monte Carlo simulations to obtain the best estimation for the densities of the aggregates.

3 $$\rho_{agg} = \frac{3}{4}\frac{{C_{d} \rho_{a} V_{t}^{2} }}{{g d_{eq} }} + \rho_{a}$$

We considered the acceleration due to gravity (*g*) as a constant with value 9.81 m s^−2^. Then, C_d_ (valid for any particle shape) was calculated using Eq. (34) of Bagheri and Bonadonna^11^. They found a general correlation for estimating the average drag coefficient for freely falling solid non-spherical particles in liquid or gases. This equation is represented in Eq. (4) as a function of the following parameters: First, Newton's drag correction ${\text{k}}_{{\text{N}}}$ (Eq. (5)) where $\alpha_{2} = 0.45 + 10/\exp \left( {2.5 \log \rho^{\prime } + 30} \right)$ and $\beta_{2} = - 37/\exp \left( {3 \log \rho^{\prime } + 100} \right)$, where $\rho^{\prime }$ (no units) is the particle-to-fluid density ratio. Additionally, ${\text{F}}_{{\text{N}}} = {\text{ f}}^{2} {\text{ e}}\left( {\frac{{{\text{d}}_{{{\text{eq}}}}^{3} }}{{\text{L I S}}}} \right)$, where *f* and *e* are the particle flatness and elongation. Second, the Stokes' drag correction *k*_*S*_ (Eq. (6)) where ${\text{F}}_{{\text{S}}} = {\text{ f}}^{2} {\text{ e}}^{1.3} \left( {\frac{{{\text{d}}_{{{\text{eq}}}}^{3} }}{{\text{L I S}}}} \right)$. Finally, the Reynolds number Re.

4 $$C_{d} = k_{N} \left( {\frac{{24k_{S} }}{{Re k_{N} }}\left( {1 + 0.125\left( {Re k_{N} / k_{S} } \right)^{2/3} } \right) + \frac{0.46}{{1 + 5330/\left( {Re k_{N} / k_{S} } \right)}}} \right)$$

5 $${\text{k}}_{{\text{N}}} = 10^{{\alpha_{2} \left[ { - \log \left( {F_{N} } \right)} \right]\beta_{2} }}$$

6 $${\text{k}}_{{\text{S}}} = (F_{S}^{1/3} + F_{S}^{ - 1/3} {)/2}$$

### Analysis of aggregates collected on adhesive tape for SEM analysis

We collected the falling aggregates on adhesive tape at the same time as the recording of the HSC videos. The adhesive tapes were exposed for less than one minute during fallout to capture individual aggregates. We analyzed the adhesive tapes using the Scanning Electron Microscope (SEM) at the University of Geneva, for six different eruptions (C, E, G, H, I and K).

SEM images were used for two purposes: first, to qualitatively classify the different aggregates (following the nomenclature of Brown et al.^1^ and Bagheri et al.^11^), and, second, to determine the grain size distribution (GSD) of selected aggregates. Because the process of obtaining the GSD is very time consuming, we selected three representative aggregates of two different types (PC2 and PC3) for eruptions C, H and K.

#### Grain size analysis of SEM images

For the selected aggregates we captured close-up images of their components and then we manually altered the images in terms of light and contrast for better visualization of the particles. Images taken from aggregates of eruptions C, H and K had magnifications of × 430, × 330, and × 370, respectively. Afterwards the images were analyzed using the software Adobe Photoshop to contour each particle composing the aggregates with the help of the magic wand tool. Once all the single particles were selected, we separated and positioned them in a way that they were not overlapping one to each other. The images were converted into binary images which are processed in Fiji using a macro based on the work of Liu et al.^41^ to calculate the Feret diameter of all the particles and to obtain the frequency number per phi (Φ) category for the aggregates. Next, we converted the number distribution into a weight percent (wt%) distribution. To do that, we used the total number of particles for each phi range (Φi) and multiplied this number by the density and the volume to find the total mass associated with the i-th bin. The density is considered as constant and independent of the particle size and equals to 2500 kg m^−3^ following the results of Bagheri et al.^11^; they measured the density of Sakurajima’s tephra using a water pycnometer and obtained values between 2300–2700 kg m^−3^. The volume is approximated to the volume of a sphere (π × d^3^/6) and we considered the diameter *d* as the average diameter for each phi bin. Finally, we normalize to 100% all the values to obtain the GSD in weight percent for each aggregate. The uncertainty associated with the GSD of the aggregates is provided in section 6 of the Supplementary Material.

### Tephra collection in trays

We collected tephra samples in rectangular plastic trays with dimensions of 16.9 × 22.9 cm for a total of six eruptions. We used a set of four trays (T1, T2, T3 and CS) to collect the falling tephra. T1, T2 and T3 collected material at an interval of two minutes and the cumulative sample (CS) was used to collect the total amount of tephra during the whole fallout. The complete information of the eruptions sampled and the time of collection for each eruption is described in Table S7 (Supplementary Material). For finding the grain size analysis of tephra samples we used only the cumulative samples as the material collected using T1, T2 and T3 was not sufficient for the analysis. First, we performed a dry mechanical sieving for particles > 500 µm in diameter; particles < 500 µm in diameter were analyzed using the laser diffraction particle-size analyzer, Bettersizer S3 Plus, at the University of Geneva. This instrument gives the particle size distribution between 11 and −1.5 Φ. Additionally, the determination of the accumulation rate was made only for three eruptions (A, B and E) and was based on the amount of material collected, the area of the tray and time of collection (kg m^−2^ s^−1^).

## Supplementary Information

Supplementary Video 1.

Supplementary Video 2.

Supplementary Video 3.

Supplementary Information 1.
