# Supplementary material for: Aerodynamic characteristics and genesis of aggregates at Sakurajima Volcano, Japan
Source: Sci Rep. 2022 Feb 7;12:2044. doi: 10.1038/s41598-022-05854-z (PMC8821550; doi:10.1038/s41598-022-05854-z)
Supplement: Supplementary file 4 — Supplementary Information 1. [file 41598_2022_5854_MOESM4_ESM.docx]

**Supplementary information for: Aerodynamic characteristics and genesis of aggregates at Sakurajima Volcano, Japan**

Diaz Vecino M.C.^1^, Rossi E.^1^, Freret-Lorgeril V.^1^, Fries A.^1^, Gabellini P.^2^, Lemus J.^1^, Pollastri S.^1^, Poulidis A. P.^3,4^, Iguchi, M.^4^, Bonadonna C.^1^

^1^ Département des Sciences de la Terre, Université de Genève, Geneva, Switzerland

^2^ Dipartimento di Scienze della Terra, Università di Firenze, Florence, Italy

^3^ Institute of Environmental Physics, University of Bremen, Bremen, Germany

^4^Disaster Prevention Research Institute, Kyoto University, Kagoshima, Japan

Corresponding author: [costanza.bonadonna@unige.ch](mailto:costanza.bonadonna@unige.ch)

1. **GPS coordinates and sampling information**

During the field sampling, data were collected at six different locations, here labelled with ‘L’ codes and the GPS coordinates, geoid used is WGS84. (Table S1).

| Location | Latitude | Longitude |
| --- | --- | --- |
| L1 | 31.5740429 | 130.7081752 |
| L2 | 31.5551946 | 130.6790334 |
| L3 | 31.6229716 | 130.6501668 |
| L4 | 31.5531824 | 130.6590439 |
| L5 | 31.5898848 | 130.6013455 |
| L6 | 31.61922222 | 130.6894444 |

**Table S1.** The ‘L’ codes are used for the sampling locations of Fig. 1. GPS coordinates are displayed in decimal format

1. **Model domain and setup options for the Weather Research and Forecasting (WRF).**

Two domains were used to downscale JMA MesoScale Model (MSM) data [1]. From the original grid spacing (Δx=10 km) progressively to 1.5 (D1) and 0.5 km (D2), following a similar methodology to Poulidis and Iguchi [2] and Poulidis *et al*. [3], which was shown to provide accurate results over Sakurajima. Model domain and setup options are shown in Tables S2 and S3 respectively. Data were output every 20 minutes and profiles shown in Fig. 6 were based on an average of two output steps.

| D | *nx* | *ny* | *nz* | *Δx* (m) | *H_TOP_* (km asl) | Longitude | Latitude |
| --- | --- | --- | --- | --- | --- | --- | --- |
| 1 | 238 | 238 | 57 | 1500 | 16.8 | 129.048-132.237 | 29.694-33.453 |
| 2 | 237 | 237 | 57 | 500 | 16.8 | 130.016-131.269 | 31.041-32.104 |

**Table S2**. WRF model domain description. Note that in the case of the WRF domain the model top is set at 10 hPa; the value in kilometers is an approximation.

| Scheme | Option | Reference |
| --- | --- | --- |
| Boundary Layer | Shin-Hong | Shin and Hong [4] |
| Microphysics | Kessler | Kessler [5] |
| Radiation | RRTMG | Iacono *et al*. [6] |
| Surface Layer | MM5 | Jimenez *et al*. [7] |
| Land Surface | MM5 | Dudhia [8] |

**Table S3.** Physics options used with the WRF model.

1. **Set up and parameters extracted from HSC video analysis and units (SI) of the variables used in the equations**

The description of each parameter is summarized in Table S4. The primary measurements extracted from the HSC video analysis were the diameter and the terminal velocity, which were used to calculate the density of the aggregates. The units of the variables used in the equations are shown in Table S5.

| Parameter | Description |
| --- | --- |
| X | X (horizontal) coordinate |
| Y | Y (vertical) coordinate |
| A | Projected area |
| Feret’s diameter | The longest distance between any two points of a particle along the selection boundary, also known as maximum caliper. |
| Minimum Feret’s diameter | The minimum caliper diameter |

**Table S4.** Aggregate’s information extracted from the videos using Fiji.


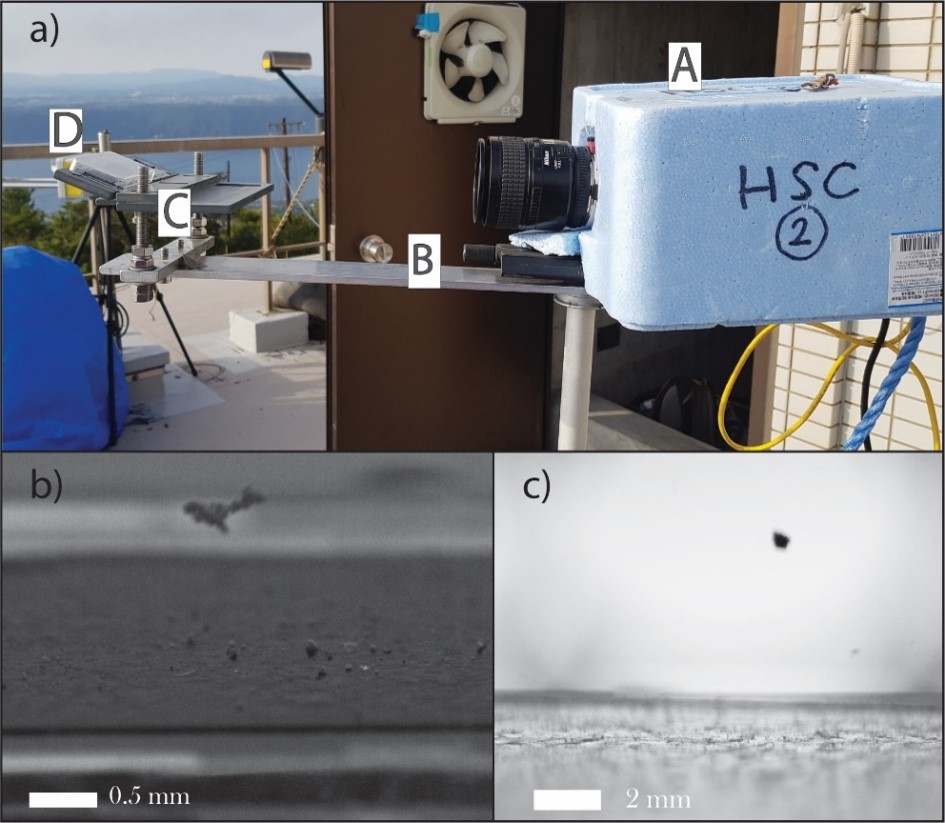


**Figure S1** (a) Set up for acquiring HSC videos and samples of falling aggregates. A is the high-speed camera with the 60 mm lens; B is the support that links the camera with the thin section; C is the support that has a 30° inclination and D is the thin section with the SEM adhesive tape covered by a plastic cap; (b) and (c) are examples of falling aggregates captured by the HSC videos.

| **Name of the variable** | **Symbol** | **Unit (SI)** |
| --- | --- | --- |
| Equivalent diameter | $d_{\mathrm{eq}}$ | μm |
| Maximum Feret diameter measured on the maximum projection of the area | L | μm |
| Minimum Feret diameter measured on the maximum projection of the area | I | μm |
| the minimum Feret diameter measured on the minimum projection of the area | S | μm |
| Terminal velocity | $\text{V}_{\text{t}}$ | m $s^{\text{-1}}$ |
| Density of the aggregate | $\text{ρ}_{\text{agg}}$ | kg$\text{m}^{\text{-3}}$ |
| Density of air | $\text{ρ}_{\text{a}}$ | kg$\text{m}^{\text{-3}}$ |
| Drag coefficient | $\text{C}_{\text{d}}$ | No units |
| gravity | g | m $s^{\text{-2}}$ |
| Particle to fluid density ratio | $\rho'$ | No units |

**Table S5**. Information about the name, symbol, and the associated units (in SI) for the main variables used in the equations.

1. **Air density for calculating the density of the aggregates**

The air density changed during the sampling days (from$\text{1.11 to 1.17 kg/}\text{m}^{\text{3}}$) and was calculated using the website for atmospheric sounding from the University of Wyoming (<http://weather.uwyo.edu/upperair/sounding.html>). The values for the air density are reported in Table S6 as well as the time of collection of the videos for the eruptions investigated.

| Eruption ID-Location | Sampling time (JST) | Air density (kg$\text{m}^{\text{-3}}$) |
| --- | --- | --- |
| B-L2 | 14:04:00 | 1.16 |
| C-L2 | 15:05:00 | 1.16 |
| D-L2 | 15:37:00 | 1.16 |
| F-L3 | 12:21:49 | 1.11 |
| G-L3 | 14:06:13 | 1.11 |
| I-L5 | 14:38:07 | 1.15 |
| K-L6 | 14:46:34 | 1.17 |

**Table S6**. Air density and sampling time for the eruptions studied with the HSC videos, sampling time (in Japan standard time (JST) format, offset UTC +9:00 hours).

1. **Tephra sampling information**

We sampled the tephra at the same locations as the HSC video and adhesive tapes were taken, although, during some eruptions we just collected tephra with no HSC videos associated (e.g., eruption A). The sedimentation of eruption A lasted 7 minutes at location L1. The time of sedimentation for eruption B was 19 minutes at location L2. Finally, sedimentation of eruption E lasted 13 minutes at location L2.

| Eruption label - type of sampling | Beginning of fallout (JST) | End of fallout (JST) | Sampling interval  (JST) | Sampling time (s) |
| --- | --- | --- | --- | --- |
| A-CS | 17:28:00 | 17:35:00 | 17:28:00-17:35:00 | 427 |
| A-T1 |  |  | 17:29:00-17:31:00 | 120 |
| A-T2 |  |  | 17:31:00-17:33:00 | 120 |
| A-T3 |  |  | 17:33:00-17:35:00 | 120 |
| B-CS | 14:03:30 | 14:23:00 | 14:03:30-14:23:00 | 1200 |
| B-T1 |  |  | 14:04:30-14:06:30 | 120 |
| B-T2 |  |  | 14:06:30-14:08:30 | 120 |
| B-T3 |  |  | 14:08:30-14:10:30 | 120 |
| E-CS | 16:20:40 | 16:33:00 | 16:20:40-16:33:00 | 780 |
| E-T1 |  |  | 16:22:00-16:24:00 | 120 |
| E-T2 |  |  | 16:24:00-16:26:00 | 120 |
| E-T3 |  |  | 16:26:00-16:28:00 | 120 |
| G-CS | 14:04:00 | 14:25:00 | 14:04:00-14:25:00 | 960 |
| I-CS | 14:28:00 | 14:44:00 | 14:28:00-14:44:00 | 960 |
| J-CS | 16:19:00 | 16:32:00 | 16:19:00-16:32:00 | 780 |

**Table S7.** Information relative to the tephra collection for every eruption, including the time of collection for the accumulation rate time (in Japan standard time (JST) format, offset UTC +9:00 hours).

1. **Uncertainties**

*Uncertainty on the diameter of the particle*

To obtain the minimum errors associated with the diameter calculation we only used HSC videos where the particles were on focus for at least 3 consecutive frames. The equivalent diameter has an uncertainty, ${\delta d}_{eq}$, described by equation (S1) and is calculated using the formula for the error propagation for mutually independent variables applied to equation (1) where the L, I and S values are explained.

| ${\delta d}_{eq}=\frac{0.928}{3}\cdot({L\cdot I\cdot S)}^{-2/3} \cdot\sqrt{{(IS)}^{2}\cdot{\delta L}^{2}+{(LS)}^{2}\cdot{\delta I}^{2}+{(LI)}^{2}\cdot{\delta S}^{2}}$ | Eq. S1 |
| --- | --- |

We obtained the uncertainty $\delta L,$ as the standard deviation of all the maximum Feret diameters for the aggregate, the uncertainty $\delta S$ is calculated using the standard deviation of the minimum Feret and for the uncertainty $\delta I$ we attributed the same value as $\delta L$. We found that the minimum relative error associated with the diameter calculation was 1.9% and the maximum relative error was 25%.

*Uncertainty on the terminal velocity*

We used the standard deviation of the velocity measurements for each aggregate to calculate the uncertainty $\delta V_{t}$. It is important to mention that the uncertainty on the velocity is also dependent on the time. However, we can assume that the chronometer of the camera is very precise for the videos and the uncertainty is, therefore, very low and negligible. The minimum and maximum relative error for the terminal velocity was 1% and 10%, respectively. The very low value of the relative error in most of the aggregates is an indication that it reached the terminal velocity before hitting the plate as we measured the standard deviation using the last 5 frames before impact.

*Uncertainty on the density*

The density of the aggregates is calculated as a non-linear relationship of some variables such as the terminal velocity, the diameter of the particle, the density of the fluid (air, in this case) and the drag coefficient that is also a function of the terminal velocity (equation (3)). Due to the non-linearity of equation (3) we applied a Monte Carlo methodology to compute the error propagation on the density of the aggregate, using Gaussian distributions for the independent variables with a standard deviation equal to their uncertainty and the mean value of the distribution equal to their estimated average value. We used $\text{10}^{\text{6}}$ random combinations using the range of values of the terminal velocity ± uncertainty of the velocity and the diameter ± uncertainty of the diameter, and we obtained a Gaussian distribution for each aggregate. The mode of this distribution is taken as the final indicator of the density for the aggregate. The uncertainty interval is bound by a lower and upper value associated with the 16^th^ and 84^th^ percentile of the distribution, respectively.

*Uncertainty on the GSD of the SEM images*

There are two sources of uncertainties associated with the GSD of the images obtained with the SEM. The first one is the superposition that is when the particles that are part of the aggregate can overlap with each other and therefore, we do not account for the total amount of particles that compose the aggregate because we do not have full visibility of them when contouring in Photoshop. The second type of uncertainty is associated with the resolution of the images. As we look closer, some particles are smaller than 5 µm (Fig. S2) and we observe a decrease in the image resolution and a loss of sharpness. Hence, contouring such particles to determine their size and shape remains highly challenging. Consequently, we underestimate the number of particles belonging to Φ classes > 8. This problem comes from the acquisition of the images as ash does not stick strongly to the tape and can move when the image is being taken. For the reason mentioned before it is not possible to obtain images with greater magnification.


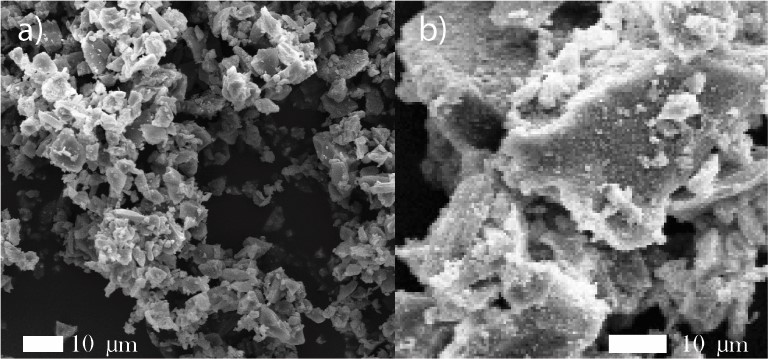


**Figure S2** Representation of the limitations of calculating the GSD from image analysis. (a) In this image we can see the overlapping of particles that compose the aggregates. (b) we observe the presence of very small particles that are difficult to characterize due to the resolution

**References**

1. Saito, K. et al. The operational JMA nonhydrostatic mesoscale model. *Monthly Weather Review*. **134**, 1266-1298; 10.1175/MWR3120 (2006).
2. Poulidis, A. P., & Iguchi, M. Model sensitivities in the case of high-resolution Eulerian simulations of local tephra transport and deposition. *Atmospheric Research*. **247**, 105136; 10.1016/j.atmosres.2020.105136 (2021).
3. Poulidis, A. P., Shimizu, A., Nakamichi, H., & Iguchi, M. A computational methodology for the calibration of tephra transport nowcasting at Sakurajima volcano, Japan. *Atmosphere*. **12**, 104; 10.3390/atmos12010104 (2021).
4. Shin, H. H., & S.-Y. Hong. Representation of the subgrid-scale turbulent transport in convective boundary layers at gray-zone resolutions. *Monthly Weather Review*. **143**, 250-271; 10.1175/MWR-D-14-00116.1 (2015).
5. Kessler, E. On the distribution and continuity of water substance in atmospheric circulations. *American Meteorological Society.* 32 ,1-84 ;10.1007/978-1-935704-36-2_1 (1969).
6. Iacono, M. J et al. Radiative forcing by long–lived greenhouse gases: Calculations with the AER radiative transfer models. *Journal of Geophysical Research: Atmospheres.* **113**, D13103; 10.1029/2008JD009944 (2008).
7. Jimenez, P.A et al. A revised scheme for the WRF surface layer formulation. *Monthly Weather Review*. **140**, 898–918. 10.1175/MWR-D-11-00056.1 (2012).
8. Dudhia, J. A multi-layer soil temperature model for MM5. the Sixth PSU/NCAR Mesoscale Model Users' Workshop. 22-24 (1996).
